# Combining ability and testcross performance of multi-nutrient maize under stress and non-stress environments

**DOI:** 10.3389/fpls.2023.1070302

**Published:** 2023-01-24

**Authors:** Nakai Matongera, Thokozile Ndhlela, Angeline van Biljon, Casper N. Kamutando, Maryke Labuschagne

**Affiliations:** ^1^ Scientific and Industrial Research and Development Centre (SIRDC), Harare, Zimbabwe; ^2^ Global Maize Program, International Maize and Wheat Improvement Centre (CIMMYT), Harare, Zimbabwe; ^3^ Department of Plant Sciences, University of the Free State, Bloemfontein, South Africa; ^4^ Department of Plant Production Sciences and Technologies, University of Zimbabwe, Harare, Zimbabwe

**Keywords:** malnutrition, multi-nutrient maize, zinc-enhanced, combining ability, abiotic stress, drought, nitrogen

## Abstract

While significant progress has been made by several international breeding institutions in improving maize nutritional quality, stacking of nutritional traits like zinc (Zn), quality protein, and provitamin A has not received much attention. In this study, 11 newly introduced Zn-enhanced inbred lines were inter-mated with seven testers from normal, provitamin A and quality protein maize (QPM) nutritional backgrounds in order to estimate the general combining ability (GCA) and specific combining ability (SCA) for grain yield (GY) and secondary traits under stress conditions [(combined heat and drought stress (HMDS) and managed low nitrogen (LN)] and non-stress conditions [(summer rainfed; OPT) and well-watered (irrigated winter; WW)] in Zimbabwe. Lines L6 and L7 had positive GCA effects for GY and secondary traits under OPT and LN conditions, and L8 and L9 were good general combiners for GY under HMDS conditions. Superior hybrids with high GY and desirable secondary traits were identified as L10/T7 and L9/T7 (Zn x normal), L2/T4, L4/T4, L3/T5 (Zn x provitamin A), and L8/T6 and L11/T3 (Zn x QPM), suggesting the possibility of developing Zn-enhanced hybrids with high yield potential using different nutritional backgrounds. Both additive and dominance gene effects were important in controlling most of the measured traits. This suggests that selecting for desirable traits during inbred line development followed by hybridization and testing of specific crosses under different management conditions could optimize the breeding strategy for stacked nutritionally-enhanced maize genotypes.

## Introduction

Global cereal demand for human consumption and livestock feed is rapidly increasing due to continuous population growth (Alexandratos and Bruinsma, 2012). Among cereals, maize demand is the highest, followed by wheat and rice, which together account for 94% of all cereal consumption (Ranum et al., 2014). The increased maize demand will have a great impact on the low and middle-income countries such as those in sub-Saharan Africa (SSA) and south Asia with an undernourishment prevalence of 22.8% and 14.7%, respectively (Abegaz, 2018). Besides food insufficiency, several millions of people in these low and middle-income regions suffer from micronutrient deficiencies such as Zn and vitamin A, as well as essential amino acids such as lysine and tryptophan (Nair, 2020; Nkhata et al., 2020). One of the reasons for this is overreliance on maize-based foods with limited supplementary foods, which exposes a high proportion of the population to the risk of micronutrient and protein deficiency (Prasanna et al., 2001; Nuss and Tanumihardjo, 2011). Vitamin A and Zn deficiency negatively affects the physical well-being, eyesight and cognitive development of people and can lead to birth defects and child mortality (Menkir, 2008; Rautiainen et al., 2016). Tryptophan is a precursor amino acid for niacin (vitamin B3) biosynthesis, and therefore its deficiency causes pellagra (Gupta et al., 2015; Kipsang et al., 2019). General lack of dietary protein in weaned infants adversely affects their overall well-being, and symptoms include peripheral oedema, diarrhea and severe wasting, collectively known as “kwashiorkor” (Goredema-Matongera et al., 2021).

To meet both nutritional and high maize demand, several studies have suggested the need to develop multi-nutrient biofortified maize hybrids with high yield potential (Ray et al., 2013). This breeding initiative could have huge benefits to maize-based low-income societies such as in SSA. Breeding for high yielding biofortified hybrid maize is a cost-effective and sustainable strategy to increase both maize productivity and nutritional quality. Hybrid maize is developed by crossing two or more parents to exploit maximum heterosis. Whilst hybrids generally have high yield potential, developing stable biofortified hybrids with outstanding performance under both stress and non-stress environments is a key priority in SSA (Miranda et al., 2008). Most of the cropping systems in SSA are characterized by various abiotic stress factors that threaten food security and humanity in this region and these include low soil nitrogen (Makumbi et al., 2011), and combined heat and drought stress (Cairns et al., 2013; Meseka et al., 2018). The incidence of drought and heat stress and the continuous decline in soil fertility and water holding capacity is expected to increase due to global climate change (Ertiro et al., 2017). Projections are that by 2030, 40% of the arable land will be unsuitable for the maize varieties grown currently (Meseka et al., 2018). The simultaneous occurrence of heat and drought stress in the tropical lowlands is likely to increase and has greater adverse effects on agronomic traits than when each stress occurs separately (Cairns et al., 2013; Bhardwaj et al., 2021). In addition, the intensified crop production in SSA, monocultural practices and growing crops in marginal areas, have contributed significantly to the rapid decline of soil fertility in SSA (Makumbi et al., 2011). Therefore, strong adaptation measures such as developing multiple stress tolerant maize are required to enable small-holder farmers to cope with these production constraints (Salami et al., 2010). Breeding for nutritionally superior hybrids that can withstand multiple stress conditions is an attractive strategy to mitigate both food insecurity and several nutritional challenges experienced in SSA simultaneously (Chakraborti et al., 2009; Hindu et al., 2018).

While concerted efforts are being made towards developing biofortified maize hybrids, breeding for Zn-enhanced hybrid cultivars is lagging behind (Garcia-Oliveira et al., 2018). The International Maize and Wheat Improvement Centre (CIMMYT) and its partners have initiated breeding programs for Zn-enhancement in maize (Goredema-Matongera et al., 2021). Wide genetic variation for Zn exists in normal maize (Garcia-Oliveira et al., 2018), QPM (Chakraborti et al., 2009; Hindu et al., 2018) and provitamin A maize (Hoisington, 2002). Improving maize kernel Zn content in maize already biofortified for provitamin A and QPM increases its overall nutritional composition (Maziya-Dixon et al., 2000; Prasanna et al., 2020). In this way, millions of lives could be saved from various macro- and micro-nutrient deficiencies and this concurs with the agenda for the United Nation’s Sustainable Development Goals for 2030 to end hunger in all its forms (Gil et al., 2019).

To develop highly productive multi-nutrient maize hybrids, breeders should know the GCA and SCA of inbred lines, since they both indicate the breeding value of inbred lines in hybrid combinations (Karim et al., 2018). GCA has been defined by Sprague and Tatum (1942) as the average performance of the inbred line based on its performance in crosses with other inbred lines. SCA refers to the performance of an inbred line in a specific cross. Combining ability is a powerful tool for identifying the best performing inbred lines for use in different cross combinations either to exploit heterosis or accumulate fixable genes (Uddin et al., 2008). This reveals the mode of gene action for a particular trait. GCA is associated with additive gene effects, whereas SCA is associated with non-additive effects including dominance and epistatic gene effects (Aguiar et al., 2003). Several studies evaluated the combining ability of maize inbred lines grown under managed drought stress (Bänziger et al., 2006; Derera et al., 2007), drought and low N stress (Makumbi et al., 2011; Beyene et al., 2013), optimum conditions (Ertiro et al., 2017) and heat stress (Archana et al., 2018). Despite all this, studies on combining ability for GY for biofortified maize inbred lines and testcross performance under stress and non-stress conditions are still limited. Such studies are useful to breeders pursuing breeding for quality traits using exotic nutrient donors that introgress desired nutritional traits into local germplasm pools. Therefore, the objective of this study was to estimate the GCA and SCA of introduced Zn donors in testcrosses involving normal, provitamin A and QPM testers, under WW, managed low N and HMDS conditions.

## Materials and methods

### Plant materials

Eleven Zn-enhanced inbred lines (zinc donors) from CIMMYT and International Institute of Tropical Agriculture (IITA) were crossed with seven testers from normal, provitamin A and QPM nutritional background and the mating scheme resulted in 77 single-cross hybrids (**Table 1**). Zn donors were evaluated in CIMMYT-Mexico and Zimbabwe primarily for kernel Zn content in previous studies (unpublished data) and the means are presented in **Table 1**. Similarly, the testers were screened and selected for comparably high Zn content than other inbred lines in the respective breeding programs. Provitamin A and QPM testers (**Table 1**) used in this study have been widely used as nutrient donors at CIMMYT because of relatively high β-carotene (> 12 µg/g) and tryptophan (>0.8%) content respectively.

**Table 1:** Description of the plant materials used for making crosses.

NO.	Code	Role	Nutritional profile	Zinc content (mg kg^-1^)	Heterotic group^ǂ^	Source
**1**	L1	Line	Zinc	30.02	B	IITA
**2**	L2	Line	Zinc	30.09	B	IITA
**3**	L3	Line	Zinc	27.25	A	IITA
**4**	L4	Line	Zinc	34.29	A	IITA
**5**	L5	Line	Zinc	30.25	A	IITA
**6**	L6	Line	Zinc	33.85	A	CIMMYT-Mexico
**7**	L7	Line	Zinc	33.72	A	CIMMYT-Mexico
**8**	L8	Line	Zinc	30.36	B	CIMMYT-Mexico
**9**	L9	Line	Zinc	28.68	B	CIMMYT-Mexico
**10**	L10	Line	Zinc	32.18	A	CIMMYT-Mexico
**11**	L11	Line	Zinc	30.52	B	IITA
**12**	T1	Tester	Normal	34.39	A	CIMMYT-Zimbabwe
**13**	T2	Tester	Normal	28.34	AB	CIMMYT-Zimbabwe
**14**	T3	Tester	QPM	35.48	A	CIMMYT-Zimbabwe
**15**	T4	Tester	Provitamin A	28.10	A	CIMMYT-Zimbabwe
**16**	T5	Tester	Provitamin A	30.82	B	CIMMYT-Zimbabwe
**17**	T6	Tester	QPM	29.19	B	CIMMYT-Zimbabwe
**18**	T7	Tester	Normal	30.11	B	CIMMYT-Zimbabwe

^ǂ^Heterotic group classification: Group A = Tuxpeno, B73 types; Group B = Eto, Ecuador, and Mo17 types; IITA, International Institute of Tropical Agriculture; QPM, Quality Protein Maize.

### Experimental design and trial management

The generated 77 line x tester crosses were evaluated together with seven commercial hybrids in 10 location-year combinations under four optimum (summer rainfed, OPT), two well-watered or winter irrigated (WW), two combined heat and drought stress (HMDS) and two managed low N (LN) management conditions in Zimbabwe (**Table 2**). The 84 hybrids were laid out across all the 10 locations using an alpha (0.1) lattice design. Evaluation trials under LN and HMDS were grown in well-established screening sites that have been developed for routine testing for maize germplasm. LN sites were established by continuously growing maize during the main season or irrigated wheat in the winter dry season for more than 10 years with subsequent removal of all crop residues after harvesting. Nitrogenous fertilizers were not applied for top dressing at LN sites, but instead, Muriate of Potash (MOP) and single super phosphate (SSP) fertilizers were applied to provide the crop with adequate potassium and phosphorus, respectively.

**Table 2:** Description of testing environments used for this study.

Location	Year	Season	Latitude	Longitude	Altitude (masl)	Management
**CIMMYT**	2019/20	Summer	17°48’ S	31°03’ E	1483	Optimum
**CIMMYT**	2019/20	Summer	17°48’ S	31°03’ E	1483	Low N stress
**RARS**	2019/20	Summer	17°48’ S	31° 3’ E	1369	Optimum
**DR&SS**	2019/20	Summer	17°13’ S	31°03’ E	1506	Low N stress
**Gwebi**	2019/20	Summer	17°41’ S	30°32’ E	1448	Optimum
**ART farm**	2019/20	Summer	17°42’ S	31° 5’ E	1556	Optimum
**Chiredzi**	2020	Winter	21°02’ S	31°57’ E	433	Drought stress
**Chiredzi**	2020	Winter	21°02’ S	31°57’ E	433	Well-watered
**Chisumbanje**	2020	Winter	20°47’ S	32°13’ E	480	Drought stress
**Chisumbanje**	2020	Winter	20°47’ S	32°13’ E	480	Well-watered

masl, meter above sea level.

### Data collection and statistical analysis

Plant height (PH) measurements were taken at mid anthesis as the distance from the ground surface to the node bearing the flag leaf. A laser distance meter was used to measure all the plants in the plot and recording an average. Number of days to anthesis (AD) was recorded per plot when half of the plants had tassels that shed pollen. Silking date was also recorded when 50% of the plants had protruding silks. The difference between the number of days to silking and anthesis was recorded as the anthesis silking interval (ASI). At harvesting, the number of ears per plant (EPP) was determined as the proportion of the total number of ears harvested per plot divided by the total number of plants. Grain yield (GY) was recorded per plot and adjusted to 12.5% moisture content. GY was only measured for the net plot area as the two border plants close to the alley were discarded. Micronutrient analysis (grain Zn) for hybrids was done using atomic absorption spectrometry as described by Zarcinas et al. (1987).

Analysis of variance (ANOVA) for the measured traits for individual and across sites was performed using Multi-Environment Trial Analysis with R (META-R) (Alvarado et al., 2015). Differences of genotype means within each management type were determined using the least significant difference (LSD) procedure at 5% significance level (Vidotti et al., 2019). Genotypic correlations among the sites in terms of GY were calculated using META-R. This program generates correlations by calculating distance matrices and producing dendrograms or environment clusters using the PROC Cluster and PROC Tree. GCA and SCA and variance components for all the traits were estimated using the Line x Tester analysis procedure that is embedded in the Analysis of Genetic Designs with R (AGD-R) (Rodríguez et al., 2015). The analysis was performed using the method developed by Kempthorne (1957), for multi-environment data observed from trials laid out in an alpha (0.1) lattice experimental design. The total sums of squares for genotypes and genotype x environment interaction were partitioned into variation due to lines and testers (GCA), line x tester (SCA), and their interactions with site or environments (GCA x environment and SCA x environment), respectively (Makumbi et al., 2011). The following statistical model was used for estimation of combining ability effects:


$$Y_{ijk}=\;µ+L_{i}+T_{j}+LT_{ij}+LE_{ie}+TE_{je}+LTE_{ije}+E_{e}+REP_{k}\left(E_{e}\right)+BLK\left(REP_{k}\;E_{e}\right)+\;\epsilon_{ijke}$$

Where: Y_ijk_ = mean trait value observed on a cross i x j in k^th^ replication, μ = grand mean, L_i_ = GCA effect of the i^th^ line, T_j_ = GCA effect of the j^th^ tester, LT_ij_ = SCA effect of the cross i x j, LE_ie_ = effect of the i^th^ line in the e^th^ environment, TE_je_ = effect of the j^th^ tester in the e^th^ environment, LTE_ije_ = effect of the cross i x j in e^th^ environment, E_e_ = effect of the e^th^ environment, REP_k_ (E_e_) = effect of k^th^ replication nested within e^th^ environment, BLK (REP_k_ E_e_) = random effect of block nested in replicate *k* nested in environment *e*, ϵ_ijke_ = error associated with each observation or experimental error.

In AGD-R, the proportion of additive and dominance variance components for grain yield and other secondary traits was computed using the using Baker’s ratio (Baker, 1978), considering that the genetic variance between single-cross progeny is 2σ^2^_A_ + σ^2^_D_ which is equivalent to addition of the mean squares contribution from the GCA and SCA (Rukundo et al., 2017). The Baker’s ratio formula used to generate variance components was:

GCA/SCA = 2σ^2^GCA/(2σ^2^GCA + σ^2^SCA).

Narrow sense heritability was estimated for each trait per single environment and across the environments for both inbred lines and single cross hybrids using the following equation:


$$h^{2}=\;\frac{\sigma_{A}^{2}}{\;\sigma_{A\;\;\;}^{2\;}+\;\sigma_{D\;\;}^{2}+\;\sigma_{E\;}^{2}}$$

Where: σ^2^_A_ = the additive genetic variance, σ^2^_D_ = the dominance genetic variance, σ^2^_E_ = environmental variance

## Results

### Analysis of variance and performance of hybrids under stress and non-stress conditions

The combined ANOVA showed significant effects (P ≤ 0.01) of genotype (G), environment (E), and G x E for GY and secondary traits under OPT, LN, and HMDS. Genotype and environmental main effects were significant for GY, but G x E was not significant under WW conditions. Genotype main effects were significant (P ≤ 0.05) for all secondary traits such as AD, ASI, PH, and EPP across all management conditions.

The average GY of trials was 8.02 t ha^–1^ under optimum conditions, 7.1 t ha^–1^ under WW, 3.2 t ha^–1^ under HMDS, and 1.1 t ha^–1^ under LN conditions (**Table 3**). Compared to OPT conditions, GY was reduced by 10%, 60% and 86% under WW, HMDS and LN conditions, respectively. The average number of days to mid-anthesis under HMDS was 65.5, OPT was 70.0, WW was 70.9, and 71.0 under LN. ASI was highest under LN conditions (3.0 days) and lowest under WW (1.1 days), followed by OPT (1.6 days) conditions (**Table 3**). Plants were the tallest under WW (227.7 cm), followed by OPT (219.2 cm) conditions and shortest under LN (184.4) and HMDS (188.9 cm) conditions. EPP was highest under OPT conditions (1.0), followed by WW (0.9), and lowest under LN (0.5) and HMDS (0.7) conditions (**Table 4**). Results from the combined analysis by management showed that hybrids L10/T7 and L9/T7 were consistently high yielding across all four management conditions. Both L10/T7 and L9/T7 are hybrids constituted from Zn donor and normal inbred lines. The average GY of the top 20 yielding Zn-enhanced experimental hybrids and the yield of the best experimental hybrid was higher than the average of all commercial checks across all management conditions (**Table 3**; **Supplementary Table 1**). The GY performance of the top 20 Zn-enhanced hybrids was 2%, 19%, 19% and 17% higher than the average of all checks under OPT, WW, HMDS, and LN conditions, respectively. Although the main focus of this study was on combining ability for grain yield and other yield related traits, grain Zn concentration of the testcrosses varied across management type and ranged from 10.7 to 57.8 mg kg^-1^ (**Supplementary Table 2**).

**Table 3:** Grain yield (t ha^-1^), days to mid-anthesis, and anthesis silking interval of the top 20 yielding hybrids under different management conditions.

Entry	GY OPT	AD OPT	ASI OPT	Entry	GY WW	AD WW	ASI WW	Entry	GY HMDS	AD HMDS	ASI HMDS	Entry	GY LN	AD LN	ASI LN
L6/T1	11.3	70.0	1.9	L5/T1	8.9	68.8	0.8	L7/T2	4.9	63.0	2.8	**L9/T7**	3.3	68.8	0.7
**L10/T7**	10.1	69.1	2.1	L2/T7	8.8	69.5	1.3	L3/T3	4.8	65.8	2.3	**L10/T7**	3.2	68.3	0.7
L2/T2	10.0	70.0	1.6	L11/T5	8.8	69.3	1.5	**L9/T7**	4.7	65.8	3.3	L7/T6	3.2	71.3	0.4
L11/T3	9.8	71.8	1.9	L6/T7	8.6	72.5	1.5	L6/T7	4.6	65.5	2.5	L8/T7	3.2	72.3	0.5
L6/T2	9.6	69.4	1.5	L6/T6	8.6	70.0	1.0	L7/T1	4.6	64.5	1.5	L8/T3	3.1	70.8	0.6
L7/T3	9.6	73.9	1.3	L8/T7	8.6	74.8	0.5	L6/T6	4.4	62.5	2.0	L8/T6	3.1	70.3	0.7
L6/T6	9.5	69.4	1.4	L2/T4	8.5	73.5	0.5	L3/T6	4.4	67.5	2.0	L7/T3	3.1	70.3	0.4
L10/T1	9.4	69.5	1.6	L3/T6	8.5	71.8	0.8	L7/T6	4.3	62.0	2.5	L6/T1	3.0	69.0	0.5
L6/T5	9.4	69.9	2.5	L6/T4	8.4	74.3	0.5	L5/T7	4.2	65.3	3.5	L9/T1	3.0	71.0	0.4
L1/T2	9.4	71.5	1.5	L1/T2	8.4	71.5	0.5	L9/T1	4.2	62.8	0.8	L4/T1	3.0	69.0	0.6
L11/T6	9.3	70.9	1.5	L9/T1	8.3	71.8	0.8	**L10/T7**	4.2	63.3	2.8	L6/T7	2.9	68.5	0.5
L7/T1	9.3	71.5	1.5	**L10/T7**	8.2	68.3	2.5	L4/T1	4.2	64.8	4.3	L6/T3	2.8	69.0	0.4
L9/T4	9.1	71.1	1.5	L3/T7	8.1	72.0	0.5	L9/T2	4.2	65.8	2.3	L7/T1	2.8	73.5	0.4
L7/T2	9.1	69.4	-0.1	L2/T5	8.1	73.3	0.3	L1/T1	4.1	68.3	1.5	L3/T1	2.7	72.3	0.4
L7/T4	9.1	69.5	1.3	**L9/T7**	8.1	71.5	1.0	L6/T4	4.0	65.8	2.5	L8/T1	2.7	72.8	0.4
**L9/T7**	9.1	70.4	0.4	L11/T4	8.1	69.0	1.0	L11/T7	4.0	64.3	4.0	L1/T1	2.5	69.0	0.6
L1/T1	9.0	70.4	1.8	L4/T2	8.0	71.5	1.3	L4/T7	4.0	66.8	2.5	L2/T1	2.4	68.3	0.5
L11/T1	8.8	68.4	1.8	L11/T1	8.0	73.0	1.0	L11/T3	4.0	66.3	0.3	L1/T2	2.4	70.3	0.4
L11/T7	8.8	69.6	1.5	L6/T1	8.0	72.3	1.5	L2/T4	3.9	67.8	0.3	L10/T1	2.3	72.0	0.5
L11/T5	8.8	70.4	1.4	L7/T7	8.0	69.8	1.5	L2/T2	3.8	65.0	3.5	L2/T5	2.2	72.8	0.5
**Grand mean**	8.0	70.0	1.6		7.1	70.9	1.1		2.5	65.5	2.7		1.9	71.0	3.0
**Locations**	4	4	4		2	2	2		2	2	2		2	2	2
**LSD**	2.3	3.7	2.5		2.1	3.2	1.6		2.4	3.2	1.8		1.1	2.6	1.8
**Heritability**	0.88	0.6	0.53		0.85	0.52	0.63		0.46	0.22	0.12		0.80	0.53	0.59
**Top 20 hybrids**	9.4				8.3				4.3				2.8		
**Mean of checks**	9.2				7.0				3.6				2.4		
**The best hybrid**	11.3				8.9				4.9				3.3		
**The best check**	12.3				8.3				4.7				3.1		

Genotypes common to all management levels are in boldface and underlined, while those common to optimum (OPT), combined heat and drought (HMDS) and managed low nitrogen (LN) are only underlined.

**Table 4:** Plant height (cm), and number of ears per plant of the top 20 hybrids under different management conditions.

Entry	PH OPT	EPP OPT	Entry	PH WW	EPP WW	Entry	PH HMDS	EPP HMDS	Entry	PH LN	EPP LN
L6/T1	240.2	1.1	L5/T1	235.8	1.0	L7/T2	182.2	0.7	**L9/T7**	204.3	0.7
**L10/T7**	226.9	1.1	L2/T7	235.3	0.9	L3/T3	190.9	0.8	**L10/T7**	186.9	0.7
L2/T2	230.9	1.0	L11/T5	231.4	0.9	**L9/T7**	199.3	0.8	L7/T6	200.3	0.4
L11/T3	225.9	1.1	L6/T7	230.5	1.0	L6/T7	185.8	0.8	L8/T7	178.1	0.5
L6/T2	234.4	1.0	L6/T6	233.4	0.9	L7/T1	193.0	0.8	L8/T3	201.6	0.6
L7/T3	234.3	1.0	L8/T7	231.6	1.0	L6/T6	194.8	0.8	L8/T6	205.3	0.7
L6/T6	231.6	1.0	L2/T4	232.1	1.0	L3/T6	207.2	0.9	L7/T3	197.8	0.4
L10/T1	219.8	1.1	L3/T6	230.9	0.9	L7/T6	189.6	0.7	L6/T1	186.1	0.5
L6/T5	236.0	1.0	L6/T4	230.9	0.9	L5/T7	191.6	0.8	L9/T1	172.9	0.4
L1/T2	223.0	1.1	L1/T2	240.3	1.0	L9/T1	196.7	0.9	L4/T1	192.5	0.6
L11/T6	226.5	1.0	L9/T1	238.5	0.9	**L10/T7**	194.6	0.7	L6/T7	181.1	0.5
L7/T1	237.7	1.0	**L10/T7**	239.4	1.0	L4/T1	196.7	0.6	L6/T3	205.5	0.4
L9/T4	222.3	1.1	L3/T7	222.0	0.9	L9/T2	190.1	0.8	L7/T1	180.4	0.4
L7/T2	219.0	1.0	L2/T5	221.8	1.0	L1/T1	195.3	0.8	L3/T1	197.6	0.4
L7/T4	227.7	1.1	**L9/T7**	219.1	0.8	L6/T4	185.4	0.8	L8/T1	170.9	0.4
**L9/T7**	231.5	1.0	L11/T4	233.1	1.1	L11/T7	204.5	0.9	L1/T1	199.2	0.6
L1/T1	227.6	0.8	L4/T2	236.4	1.0	L4/T7	203.8	0.8	L2/T1	197.3	0.5
L11/T1	222.4	1.0	L11/T1	217.6	0.9	L11/T3	179.8	0.8	L1/T2	191.9	0.4
L11/T7	220.9	1.0	L6/T1	214.0	1.0	L2/T4	206.4	0.9	L10/T1	182.2	0.5
L11/T5	211.0	1.0	L7/T7	229.3	0.9	L2/T2	180.8	0.7	L2/T5	178.1	0.5
**Grand mean**	219.2	1.0		227.0	0.93		188.9	0.7		184.4	0.5
**Locations**	4	4		2	2		2	2		2	2
**LSD**	27.6	0.3		23.7	0.2		21.9	0.2		21.9	0.3
**Heritability**	0.73	0.25		0.62	0.58		0.77	0.76		0.75	0.46

Genotypes common to all management levels are in boldface and underlined, while those common under optimum, HMDS and LN are only underlined.

### Genotypic variance, heritability and correlation

**Figure 1:**
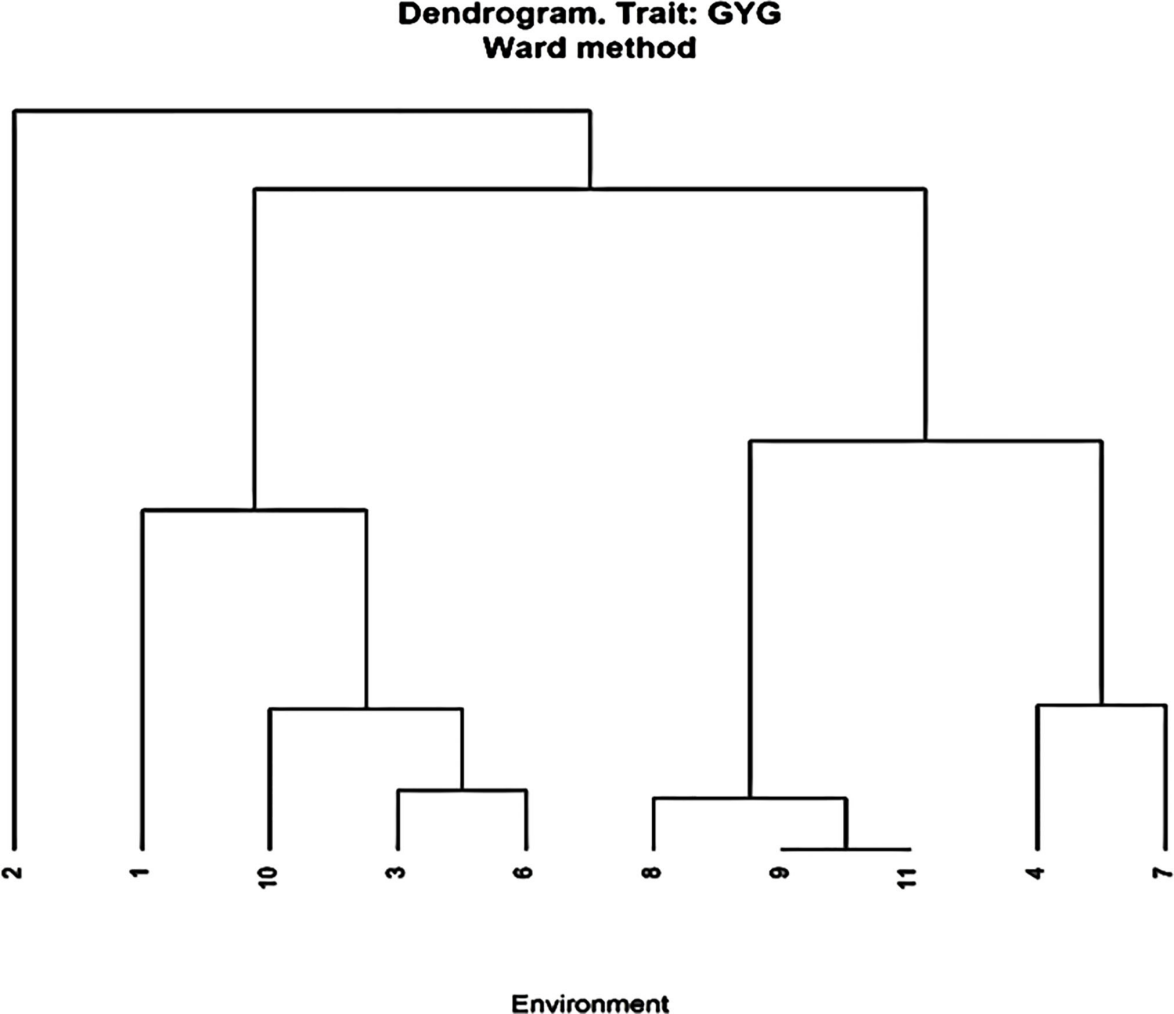
Cluster analysis of environments based on grain yield of 84 hybrids from 11 lines and seven testers and seven checks grown at 10 stress and non-stress environments in Zimbabwe. Optimum environments were 1 = ART Farm; 2 = CIMMYT; 4 = RARS; 7 = Gwebi. Managed LN environments were 3 = CIMMYT LN; 6 = DR&SS and combined heat and drought environments were 8 = Chiredzi; 10 = Chisumbanje. Well-watered environments were 9 = Chiredzi; 11 = Chisumbanje.

Genotypic variance was higher than error variance under OPT sites, LN and HMDS sites (**Table 5**). However, genotype variance was lower that the error variance in Chisumbanje under WW conditions. The average ratio of genotype to residual variance from individual experimental sites was 3.31, 0.91, 1.64, and 1.20 under OPT, WW, LN and HMDS management conditions. Sites or management levels that had higher ratio of genotype to residual variance had higher heritability for GY. The highest heritability was observed under OPT conditions, followed by LN conditions. For individual sites, the highest heritability was observed at RARS (92%) and lowest at Chisumbanje WW (54%) (**Table 5**), with an overall average heritability of 72%. Despite this, the other WW site (Chiredzi) had higher heritability of 72%, which was comparable to heritability of the two HMDS sites (**Table 5**). Cluster analysis for the environments based on GY performance of all entries showed that all LN sites clustered together, as did all WW sites, but in general, stress and non-stress environments clustered separately (**Figure 1**). Multi-location analysis by management showed that the proportion of entry or genotype variance was higher than both genotype x environment and error variances under OPT conditions than it was under stress conditions.

**Table 5:** Individual site characterization in terms of average grain yield, coefficient of variation (CV), heritability and variances of trials.

Location	Management	Year	Mean	CV (%)	Genotype variance	Residual variance	Heritability
**ART Farm**	Optimum	2019/20	7.48	22.91	9.57	2.98	0.86
**CIMMYT**	Optimum	2019/20	6.87	15.18	3.14	1.11	0.85
**CIMMYT**	Managed LN	2019/20	2.00	26.28	0.47	0.31	0.75
**RARS**	Optimum	2019/20	8.49	9.27	3.59	0.61	0.92
**DR&SS**	Managed LN	2019/20	1.83	27.46	0.47	0.26	0.78
**Gwebi**	Optimum	2019/20	9.25	7.83	1.19	0.48	0.83
**Chiredzi**	Managed Drought	2020	1.69	32.64	0.37	0.30	0.71
**Chiredzi**	Well-watered	2020	6.86	14.38	1.37	1.05	0.72
**Chisumbanje**	Managed Drought	2020	4.71	23.65	1.66	1.39	0.71
**Chisumbanje**	Well-watered	2020	7.25	15.64	0.77	1.28	0.54

Among the top yielding 20 experimental hybrids under each management level, the highest number of common hybrids (nine) was observed between OPT and HMDS conditions, followed by OPT and LN (seven) and managed HMDS and low N (seven). OPT and WW management levels had the lowest number of common hybrids (six). Among the traits, GY was highly significant and positively correlated with PH (r = 0.71^**)^, and EPP (r = 0.63^**^) under OPT conditions. A similar trend was observed in other management conditions. Under OPT conditions, ASI was weakly but positively correlated with GY (r = 0.15^**^), but this trait showed significant and negative correlation with GY under LN (r = -0.73^*^) and HMDS (r = -0.27^*^) management levels.

### Line by tester analysis

**Figure 2:**
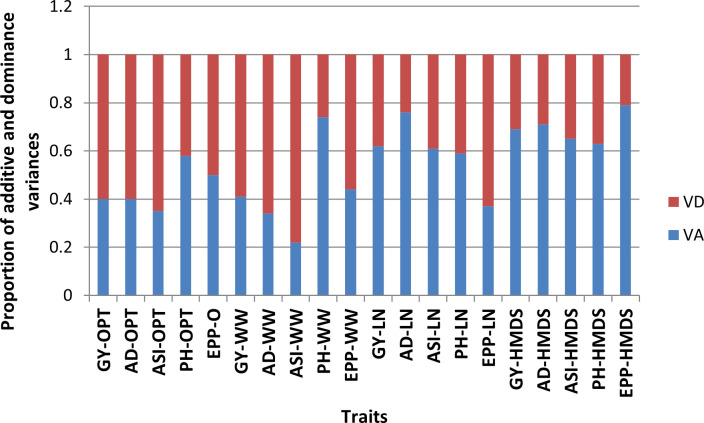
Proportion of additive (VA) and dominance (VD) variance components for grain yield and other secondary traits under optimum (OPT), well-watered (WW), managed low nitrogen (LN) and heat and drought (HMDS) conditions.

Variation due to genotype was significant (P ≤ 0.05) for most of the traits across all four management levels except for ASI under OPT conditions (**Table 6**). Line and tester mean squares were significant (P ≤ 0.05) for all measured traits except for ASI under OPT conditions. Genotypic variation due to line x tester interaction was significant across all management levels except for ASI under OPT, and PH under LN conditions (**Table 6**). Mean squares for genotype by site, line by site and tester by site interactions varied in terms of significance across sites. Site-by-line-by-tester interaction mean squares were not significant for ASI under OPT conditions, GY, PH and EPP under WW conditions, EPP under LN, and PH and EPP under HMDS conditions. Under OPT and WW conditions, the proportional contribution of dominance variances was more important for GY and secondary traits except for PH and EPP (**Figure 2**). For EPP, equal proportional contribution of the genetic variance components was observed. However, the contribution of additive variance was significant and important for GY and most of the secondary traits under both stress conditions except for EPP under LN.

**Table 6:** Analysis of variance of F_1_ crosses for grain yield and secondary traits under different management conditions.

	Optimum (Summer-rain-fed)						Well-watered (Winter-irrigated)					
Source of variation	DF	GY	AD	ASI	PH	EPP	DF	GY	AD	ASI	PH	EPP
**Site**	3	110.61^*^	313.32^*^	12.99	32540.78^*^	0.83^*^	3	9.15^***^	224.57^*^	19.75^*^	453.51	1.62^*^
**Replication (Site)**	4	2.12	7.23	1.41	439.76	0.16^*^	4	0.00	9.73^**^	1.06	426.50	0.01
**Genotypes**	76	10.82^*^	19.24^*^	2.09	990.69^*^	0.05^*^	76	6.37^**^	18.79^*^	1.38^*^	603.63^*^	0.06^*^
**Line**	10	23.93^*^	14.38^*^	2.92	2571.11^***^	0.10^*^	10	8.75^*^	36.39^*^	2.14^*^	573.14^*^	0.06^*^
**Tester**	6	22.19^*^	65.67^*^	1.59	3069.16^*^	0.09^*^	6	13.21^*^	54.34^*^	0.89	555.19^*^	0.08^*^
**Line x tester**	60	7.50^*^	15.43^*^	2.00	520.03^*^	0.04^*^	60	5.29^*^	12.31^*^	1.30^*^	613.55^*^	0.06^*^
**Site x genotypes**	228	8.00^*^	10.66^*^	1.94	494.48^*^	0.05^*^	228	0.34	16.07^*^	1.18^*^	117.48	0.02
**Site x line**	30	12.05^*^	8.35^*^	2.65^**^	662.20^*^	0.06^*^	30	0.29	27.42^*^	1.75^***^	128.69	0.03^**^
**Site x tester**	18	8.09^*^	20.76^*^	1.54	520.21^*^	0.04	18	0.39	33.29^*^	1.66^***^	40.96	0.01
**Site x line x tester**	180	7.31^*^	10.04^*^	1.86	463.95^*^	0.04^*^	180	0.34	12.45^*^	1.04^**^	123.26	0.02
**Residuals**	215	1.29	3.22	1.62	182.55	0.03	215	1.10	2.25	0.68	135.91	0.01
	Managed low nitrogen						Combined heat and drought stress					
Source of variation	DF	GY	AD	ASI	PH	EPP	DF	GY	AD	ASI	PH	EPP
**Site**	3	0.56	0.00	0.91	690.00^*^	0.22^*^	3	98.88^*^	2.22	19.75^*^	10543.84^*^	0.05
**Replication (Site)**	4	0.10	1.19	0.97	1265.12^*^	0.03	4	2.20	20.07^*^	4.45^*^	970.90^*^	0.05^**^
**Genotypes**	76	1.92^*^	14.90^*^	6.27^*^	603.79^*^	0.05^*^	76	3.45^*^	11.01^*^	4.03^*^	573.15^*^	0.09^*^
**Line**	10	2.30^*^	9.23^*^	6.41^*^	1248.58^*^	0.07^*^	10	4.03^*^	15.01^*^	4.23^*^	1071.41^*^	0.13^*^
**Tester**	6	5.21^*^	52.04^*^	21.80^*^	1588.67^*^	0.13^*^	6	5.76^*^	5.36^**^	2.51^**^	1172.38^*^	0.16^*^
**Line x tester**	60	1.52^*^	12.13^*^	4.70^*^	397.84	0.04^*^	60	3.13^*^	10.91^*^	4.15^*^	429.65^*^	0.08^*^
**Site x genotypes**	228	0.35^**^	8.46^*^	2.49^*^	225.28^*^	0.01	228	1.88^*^	9.75^***^	3.61^*^	117.26	0.02
**Site x line**	30	0.32	7.47^*^	2.36^***^	200.96	0.01	30	2.55^*^	20.32^*^	4.42^*^	214.98^**^	0.02
**Site x tester**	18	0.39	5.54^***^	2.00^**^	81.33	0.01	18	0.56^*^	12.89^*^	3.09^***^	169.73	0.01
**Site x line x tester**	180	0.36^**^	8.92^*^	2.56^*^	243.73^*^	0.02	180	1.91^*^	7.68^*^	3.53^*^	95.73	0.01
**Residuals**	215	0.24	1.76	0.83	112.00	0.02	215	0.78	2.26	0.74	89.29	0.01

*P ≤ 0.05; **P ≤ 0.01; ***P ≤ 0.001; DF, degrees of freedom; GY, grain yield; AD, anthesis date; ASI, anthesis silking interval; PH, plant height; EPP, number of ears per plant.

### General combining ability

GCA effects of lines and testers were generally not consistent across the different management levels or traits (**Tables 7**, **8**). There was no inbred line that was consistently high yielding across all management levels. However, L6 and L7 had high positive GCA effects for GY under OPT, WW, and LN conditions. Despite this, both inbred lines had negative GCA effects for GY under HMDS conditions, indicating poor adaptation to heat and moisture stress conditions. Some lines such as L8 and L9 had high GCA effects for GY under both LN and HDMS conditions, suggesting the possibility of developing Zn-enhanced hybrids that can withstand these harsh growing conditions. While positive GCA effects for GY were observed for L11 under OPT, WW and HMDS conditions, this line contributed negative GCA effects for GY under LN conditions. Contrary to this, L2 and L3 had negative GCA effects for GY under OPT and stress conditions (**Tables 7**, **8**). Inbred line L4 showed negative GCA effects for GY under all management levels except for HMDS conditions. L6 and L7 had positive GCA effects for GY as well as desirable secondary traits such as ASI and EPP under OPT and LN conditions. In fact, L6 had the highest GCA effects for GY under LN, coupled with desirable shorter AD and ASI, as well as positive GCA effects for PH and EPP under these stress conditions. In terms of contribution towards GY under stress conditions, L11 showed contrasting effects compared to L6 and L7, since this line contributed positive GCA effects under HMDS as well as under non-stress conditions. Moreover, this line showed the highest negative GCA effects for ASI, highest positive PH effects and considerable effects for EPP under HMDS conditions.

**Table 7:** Estimates of lines and tester GCA effects for grain yield and other agronomic traits under optimum and well-watered conditions.

	Optimum					Well-watered				
A. Lines	GY	AD	ASI	PH	EPP	GY	AD	ASI	PH	EPP
**L1**	-0.05	0.06	-0.02	-3.83	-0.01	-0.13	0.42	0.07	5.05	0.01
**L2**	-0.08	-0.36	0.20	-2.31	0.02	0.74	-0.55	-0.43	4.29	0.07
**L3**	-0.02	-0.78	0.40	2.16	0.01	0.25	-1.80	0.10	1.73	0.01
**L4**	-1.29^*^	0.35	-0.12	1.72	-0.08	-0.92	-1.62	0.32	-10.00	-0.06
**L5**	-0.85	0.78	0.20	-2.57	-0.06	-0.47	-0.72	0.39	-5.36	-0.03
**L6**	1.02	-0.84	-0.02	-11.11^*^	0.02	0.58	1.35	-0.22	3.74	0.02
**L7**	0.31	0.58	-0.35	-8.36	0.02	0.31	1.88	-0.11	2.89	0.03
**L8**	-0.21	0.08	-0.10	14.41	-0.04	-0.21	0.53	-0.11	0.35	0.00
**L9**	-0.03	0.37	-0.34	6.87	0.03	-0.04	0.70	-0.40	-1.30	0.01
**L10**	0.24	-0.33	0.15	2.18	0.01	-0.76	-0.05	0.25	0.71	-0.09
**L11**	0.93	0.10	0.00	0.27	0.06	0.66	-0.15	0.14	-2.09	0.02
**GCA SE**	0.62	0.48	0.22	6.46	0.04	0.53	1.09	0.26	4.31	0.04
B. Testers										
**T1**	0.57	-0.16	0.05	5.43	0.02	0.62	-1.12	0.19	2.17	0.04
**T2**	0.76	-0.31	0.08	7.14	0.02	0.20	-0.71	-0.16	2.14	0.01
**T3**	-0.54	1.10	-0.23	0.29	-0.04	-0.60	1.34	-0.18	-1.76	-0.05
**T4**	-0.15	-0.28	0.15	-2.06	0.01	0.16	-1.12	-0.04	-2.88	0.01
**T5**	-0.49	-1.03	0.05	-8.64	-0.01	-0.51	0.31	0.12	-2.65	-0.06
**T6**	-0.35	1.31	0.05	-6.35	-0.04	-0.53	1.56	0.12	-3.18	0.00
**T7**	0.19	-0.62	-0.15	3.82	0.03	0.67	-0.28	-0.04	6.16	0.05
**GCA SE**	0.46	0.80	0.12	5.46	0.03	0.51	1.03	0.13	3.29	0.04

*P ≤ 0.05; GY, grain yield; AD, anthesis date; ASI, anthesis silking interval; PH, plant height; EPP, number of ears per plant.

**Table 8:** Estimates of lines and tester GCA effects for grain yield and other agronomic traits under managed low N and combined heat and drought conditions.

	Managed low nitrogen					Combined heat and drought stress				
A. Lines	GY	AD	ASI	PH	EPP	GY	AD	ASI	PH	EPP
**L1**	-0.07	0.37	0.40	1.47	-0.02	0.01	-0.55	-0.35	7.27	0.03
**L2**	-0.23	-0.03	-0.02	0.55	-0.03	-0.17	0.00	0.07	6.08	0.06
**L3**	-0.11	0.47	0.06	-2.26	0.01	-0.06	-0.07	-0.10	1.86	0.06
**L4**	-0.35	0.69	0.07	-5.96	-0.08	0.07	0.03	0.40	-11.82	-0.11
**L5**	-0.33	0.47	0.58	-10.11	-0.05	0.34	1.08	0.22	-8.58	-0.11
**L6**	0.54	-0.88	-1.06	13.39	0.10	-0.73	0.47	-0.25	4.91	0.07
**L7**	0.24	0.33	0.14	7.53	0.00	-0.38	0.42	0.15	1.73	0.02
**L8**	0.46	-0.95	-0.73	5.18	0.06	0.36	-0.28	0.43	-3.20	-0.02
**L9**	0.10	-0.35	0.09	-2.47	0.01	0.17	-0.16	0.00	6.19	0.06
**L10**	-0.10	0.33	0.32	-5.26	0.01	-0.22	-1.71^*^	0.32	-5.07	-0.09
**L11**	-0.13	-0.45	0.17	-2.06	-0.01	0.62	0.78	-0.89	0.87	0.03
**GCA SE**	0.27	0.51	0.46	6.37	0.05	0.36	0.70	0.37	5.9	0.07
B. Testers										
**T1**	0.65	-1.77	-1.28	7.48	0.09	0.26	-0.13	-0.08	1.40	0.01
**T2**	0.01	-0.99	-0.37	2.30	0.00	0.29	0.00	-0.33	2.85	0.05
**T3**	0.06	0.60	0.44	-0.75	0.04	-0.34	-0.20	-0.02	-0.96	-0.06
**T4**	-0.41	1.37	0.80	-2.98	-0.09	0.14	0.24	0.03	-3.11	0.06
**T5**	-0.43	0.78	0.60	-11.36	-0.04	-0.61	-0.34	0.32	-8.43	-0.02
**T6**	0.05	0.28	0.21	1.45	-0.01	-0.30	-0.24	0.28	-0.94	-0.09
**T7**	0.08	-0.27	-0.40	3.85	0.00	0.56	0.66^*^	-0.20	9.35	0.06
**GCA SE**	0.32	1.00	0.65	5.56	0.05	0.33	0.32	0.22	4.8	0.05

*P ≤ 0.05; GY, grain yield; AD, anthesis date; ASI, anthesis silking interval; PH, plant height; EPP, number of ears per plant.

Among the testers, T1, T2, and T7 had positive GCA effects for GY under all management conditions (**Table 7** and **8**). Contrary to this, T6 contributed negative GCA effects for GY under all management levels except for LN conditions. T5 was the poorest tester in terms of contribution towards GY, since negative GCA effects were observed under all management conditions. In addition to GY, testers T1, T2 and T7 manifested high positive GCA effects for most of the desirable secondary traits under all the management conditions. All these testers showed negative GCA effects for ASI, and positive GCA effects for PH and EPP under both LN and HMDS stress conditions. Despite the negative GCA effects for GY and secondary traits manifested by T3 and T6, under most growing conditions, these testers showed positive GCA effects under LN. The proportion of lines and testers that had positive GCA effects for GY under LN was 36% and 71%, respectively. This indicates that tolerance to LN stress was higher in testers than in the lines. A similar trend was observed under HMDS conditions, where 55% of lines had positive GCA effects for GY as compared to a relatively higher proportion of 57% observed for testers.

### Specific combining ability

**Figure 3:**
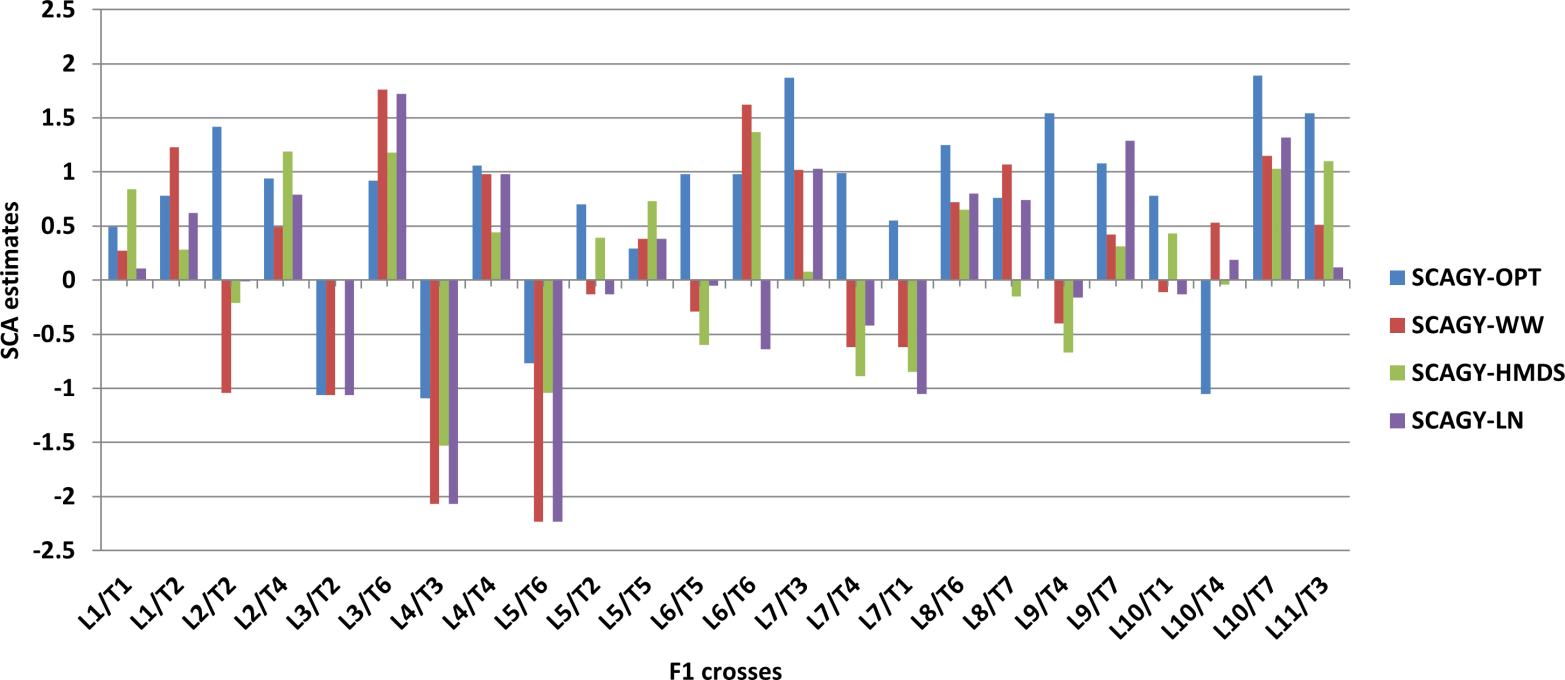
Specific combining ability (SCA) estimates of some cross combinations for grain yield (GY) under optimum, well-watered, managed heat and drought stress and low N conditions. OPT, optimum conditions; WW, well-watered; HMDS, heat and drought stress; LN, managed low N conditions.

The SCA effects of some of the best and poorest specific combiners for GY performance under different management conditions are summarized in **Figure 3**. **Table 9** depicts GY performance of the best specific combiners from different nutritional combinations. The best combiners across all management conditions were L1/T1, L1/T2, L10/T7 and L9/T7, all with a combination of Zn and normal background. Among these crosses, L10/T7 showed the highest SCA effects under both stress and non-stress environments. L2/T4, L4/T4, and L3/T6 were the best specific combiners from the Zn and provitamin A cross combinations, with good SCA effects for GY across all management conditions **Figure 3**). Similarly, L8/T6 and L11/T3 had the highest SCA effects for GY among the Zn and QPM cross combinations.

**Table 9:** Cross combinations with the highest SCA effects for grain yield under optimum, managed low N and heat and drought stress conditions.

Cross	Management	SCA effects	Mean GY (t ha^-1^)	Nutritional group
**L10/T7**	OPT	1.89	10.1	Zn + Normal
**L7/T3**	OPT	1.87	9.6	Zn + QPM
**L6/T1**	OPT	1.78	11.3	Zn + Normal
**L9/T4**	OPT	1.54	9.2	Zn + Provitamin A
**L3/T6**	LN	1.72	2.1	Zn + QPM
**L10/T7**	LN	1.32	3.2	Zn + Normal
**L9/T7**	LN	1.29	3.3	Zn + Normal
**L7/T3**	LN	1.03	3.1	Zn + QPM
**L6/T6**	HMDS	1.37	4.4	Zn + QPM
**L2/T4**	HMDS	1.19	3.9	Zn + Provitamin A
**L3/T6**	HMDS	1.18	4.4	Zn + QPM
**L10/T7**	HMDS	1.03	4.2	Zn + Normal

OPT, optimum; LN, managed low nitrogen; HMDS, combined heat and drought conditions; QPM, quality protein maize.

## Discussion

The different management conditions used in this study showed differences in the way Zn-enhanced genotypes responded to these growing conditions. Compared to the OPT conditions, GY was reduced by 60%, 86%, and 10% under HMDS, LN and WW conditions, respectively. While a very small reduction was observed under WW conditions, GY was significantly reduced under LN, followed by HMDS. The negative effect of LN and HMDS conditions on GY observed in the current study was in agreement with several previous studies (Betrán et al., 2003; Makumbi et al., 2011; Beyene et al., 2013; Ertiro et al., 2017). The average days to mid-anthesis were lowest (65.5 days) under HMDS and highest under LN (71 days). Although the number of days to mid-anthesis for OPT and WW did not vary much, HMDS and LN stress induced earlier and later flowering, respectively, compared to non-stress conditions. A similar trend was reported by Ortiz-Covarrubias et al. (2019) using provitamin A hybrids. This could have been attributed to the combined adverse effects of heat and drought stress conditions compared to heat or drought separately. Meseka et al. (2018) reported that the effects of combined heat and drought stress on normal maize are much greater than the effect of each stress separately. Therefore, early flowering observed under HMDS conditions was probably a sign of efficient utilization of the limited resources such as moisture to enable completion of the reproductive cycle (Bänziger and Lafitte, 1997). Under excessive stress conditions, most plants signal stress-induced response mechanisms, such as accumulation of high levels of abscisic acid (ABA), a hormone that regulates growth, development, dormancy, floral induction and senescence (Bruce et al., 2002; Ali et al., 2020). Therefore, high levels of ABA in plants under severe HMDS stress could signal early flowering as stress adaptive mechanisms as compared to well-watered conditions.

In addition, LN and HMDS increased ASI by 188% and 169%, respectively. WW conditions had a significant impact on ASI. Findings from previous studies of non-biofortified maize (Menkir et al., 2006; Ertiro et al., 2017), also reported an increase of ASI by 144% and 149% due to excessive moisture stress compared to optimum conditions. Therefore, a wider ASI observed in the current study under HMDS stress could be attributed to high severity of the combined stresses. High ASI was also observed under LN stress conditions. The GY reduction observed under both stress management conditions could be partly due to increased ASI, a secondary trait, which has high negative correlation with GY under stress conditions (Westgate, 1997; Ertiro et al., 2017; Meseka et al., 2018). Both LN and drought stress at flowering favors the development of male inflorescence but inhibits ear and silk development (Maiti et al., 1996; Badr et al., 2020). However, HMDS negatively affects tassel quality, pollen production and viability compared to drought alone and this ultimately negatively impacts the quality of pollen produced as well as reduction in period of pollen shedding (Cairns et al., 2012; Meseka et al., 2018). All these factors result in incomplete or nil fertilization, which ultimately increases kernel abortion, decreases kernel development, leading to fewer ears per plant causing a reduction in GY (Jacobs and Pearson, 1991; Beck et al., 1996; Magorokosho et al., 2003). ASI has been widely used for indirect selection of genotypes with high GY potential under LN, drought and HMDS conditions, by selecting genotypes with improved synchrony between the female and male flowering (Bänziger et al., 2000; Meseka et al., 2018). In addition to ASI, both LN and HMDS reduced PH and EPP compared to OPT and WW conditions. PH was reduced by 16% and 14% under LN and HMDS respectively, but did not differ much for plants grown under WW and OPT conditions. Furthermore, WW, LN and HMDS reduced EPP by 0.07%, 50% and 30%, respectively. The reduction of both PH and EPP under stress conditions observed in this study concurs with previous studies conducted on normal maize (Badu-Apraku et al., 2011; Badu-Apraku et al., 2012; Talabi et al., 2017).

The average GY of the top 20 Zn-enhanced experimental hybrids was higher than the average of all the checks under all the management conditions. In addition, slightly lower GY of the experimental hybrids than the best check demonstrates significant yield improvement of the biofortified hybrids developed by CIMMYT. In the past, biofortified maize varieties have been associated with low yield potential, and this has hindered full adoption of such nutrient-dense cultivars by farmers (Bänziger and Long, 2000; Nkhata et al., 2020). When ranking the GY performance of all the 84 hybrid entries, checks (E84) only occupied the first position under OPT conditions (**Supplementary Table 1**). This indicates that Zn-biofortified experimental hybrids performed better under WW, HMDS and LN. It is encouraging that among the commercial checks, provitamin A hybrids occupied the first position for GY performance under both stress conditions (**Supplementary Table 1**). In addition, the fourth best check hybrid under OPT conditions was E80, a QPM commercial hybrid. Although the normal checks dominated in the overall best performing check hybrids, provitamin A and QPM biofortified check hybrids also showed outstanding performance under all management conditions. These findings confirm the great progress made so far by CIMMYT to improve GY performance of tropically adapted biofortified varieties. The knowledge of the correlation between environments or traits between management levels is critical for breeders in examining similarities between environments for GY performance and secondary traits that could be useful for indirect selection under stress conditions. In this regard, the positive correlation of GY and secondary traits such as PH and EPP observed under all management conditions agrees with previous studies on normal maize reported by Badu-Apraku et al. (2011) and Talabi et al. (2017). In addition to that, GY was negatively correlated with ASI under both stress conditions, and this further confirms the effective use of ASI for indirect selection of high yielding genotypes under stress conditions reported in previous studies of normal maize (Bolaños and Edmeades, 1993; Bänziger and Lafitte, 1997; Ortiz-Covarrubias et al., 2019). Furthermore, GY was positively correlated with AD under OPT conditions and this indicates that given adequate moisture, late maturing genotypes yield more as a result of prolonged grain filling period (Edmeades et al., 1989). Clustering of environments with similar growing conditions as observed in this study was expected. Therefore, maize breeders for quality traits should develop nutrient-dense cultivars for specific environments, since the cultivars respond to these environments differently.

The most widely used breeding strategy is to select for Zn-enhanced genotypes with high yield potential under OPT conditions and then evaluate for stable performance of the selections under different stress conditions (Myers, 1985; Magorokosho et al., 2003). In this regard, L9/T7 and L10/T7 were the best Zn-enhanced experimental hybrids that combined high GY performance and stability under stress and non-stress environments. The hybrids were constituted from inbred lines with Zn and normal genetic backgrounds. These hybrids could have combined nitrogen and water-use efficiency, as well as considerable tolerance to heat stress, possibly due to heterosis or additive effects when both parents contributed favorable alleles (Makumbi et al., 2011; Ertiro et al., 2017). For instance, all the parents of L9/T7 contributed positive GCA effects for GY under LN and HMDS, but only the tester had positive GCA under non-stress conditions. Conversely, all the parents of L10/T7 showed positive GCA under OPT conditions, with the tester contributing positive GCA under stress conditions. This indicates that superior GY performance of Zn-enhanced hybrids were due to fixation of favorable genes in the respective parental inbred lines. CIMMYT and IITA usually select inbred lines for hybrid development based on desirable traits, inclusive of those indicative of stress-tolerance such as ASI, EPP, tassel blast, chlorophyll content, leaf firing and senescence (Alam et al., 2017; Meseka et al., 2018). In addition to L10/T7 and L9/T7, hybrids L2/T4, L4/T4, and L3/T5 were identified as good Zn-enhanced hybrids in a provitamin A background. Similarly, L8/T6 and L11/T3 combined high GY and desirable secondary traits across all management conditions. Favorable alleles could have been contributed by the respective parents. High GY performance of this multi-nutrient maize across management conditions demonstrates the possibility to develop multiple-stress tolerant multi-nutrient maize genotypes for commercialization in SSA.

In breeding for a particular trait, knowledge of the associated gene action is important in optimizing the breeding strategy. In the present study, the presence of both additive and non-additive gene effects was observed, confirming the complex genetic nature of GY. Dominance variances were more important for GY than secondary traits, except for PH under OPT and WW conditions. Under OPT conditions, equal proportional contribution was observed for EPP. Conversely, additive variance was significant for GY and other traits under LN and HMDS except for EPP under LN. The predominant nature of non-additive gene effects in controlling GY and secondary traits under non-stress conditions observed in the current study were also reported in previous studies but focusing on normal maize (Mhike et al., 2011; Fahad et al., 2018). Under LN and HMDS, preponderance of additive gene action was observed for GY and most secondary traits. These results are in agreement with previously studies (Derera et al., 2007; Musila et al., 2010; Ertiro et al., 2017). Despite this, knowledge on the genetic factors governing GY and secondary traits under HMDS is still limited. The presence of both additive and dominance gene action for the traits measured indicate the presence of greater genetic diversity among the parental inbred lines used to make crosses (Fahad et al., 2018). Traits controlled by additive gene effects can be effectively improved by standard selection during inbred line development and also through recurrent selection. Some Zn-enhanced hybrids showed outstanding performance under both LN and HMDS, possibly due to fixation and contribution of multiple-stress tolerant alleles from their parents. Despite the contribution of additive effects under stress conditions, dominant gene effects were important for most traits under OPT and WW conditions. This implies that the best breeding strategy would be to exploit both GCA for the parents and SCA effects for the resulting Zn-enhanced or multi-nutrient hybrids under both stress and non-stress conditions (Chiuta and Mutengwa, 2020).

The GCA effects of both lines (Zn donors) and testers were significant across all the management levels. However, the GCA effects of line x site or tester x site were only significant under OPT and HMDS conditions. This shows the need to select lines or testers that are well adapted to specific growing conditions. Comparing the lines and the testers, results show that the testers from the normal, provitamin A, and QPM genetic backgrounds were more adapted to the Zimbabwean growing conditions than the newly introduced Zn donors. Since the testers are currently used in different breeding programs at CIMMYT, their wide adaptation to both stress and adverse growing conditions reflects emphasis and breeding efforts made in breeding for multiple stress tolerance. The inbred lines with high GCA for GY and secondary traits could be used (i) in recurrent selection schemes to improve the frequency of desirable alleles for a trait in a population, (ii) as testers to evaluate newly developed or introduced Zn-enhanced inbred lines, and (iii) as parents for Zn-enhanced synthetic varieties (Makumbi et al., 2011; Mutimaamba et al., 2020). Therefore, lines with high GCA effects for GY and other important traits were identified as L6, L7 and L11 and could be useful as testers for newly developed Zn-enhanced inbred lines under stress and non-stress environments. The results of the present study demonstrate that GY potential of the Zn-enhanced normal, provitamin A and QPM hybrids can be comparable to the normal counterparts. Hence, the possibility of developing multi-nutrient stacked hybrids with high yield potential. In addition, the current study also demonstrated the possibility of identifying Zn-enhanced lines and testers from the normal, provitamin A, and QPM nutritional groups that can be useful to make multi-nutrient hybrids adapted to stress and non-stress conditions. However, further studies should focus on understanding the physiology and genetics involved in controlling the accumulation of Zn in maize kernels, as this is critical in designing an effective breeding strategy for hybrids stacked with nutrients including grain Zn. Further studies should also focus on developing Zn-enhanced inbred lines that are well adapted to a wide range of environments.

## Conclusions

Commercialization of the identified multi-nutrient hybrids could contribute significantly to both production and productivity of multi-nutrient biofortified maize in SSA. Such hybrids include L10/T7 and L9/T7 (Zn + normal), L2/T4, L4/T4, L3/T5 (Zn + provitamin A), L8/T6 and L11/T3 (Zn + QPM). Although no single Zn donor with high GCA effects for GY and other traits were identified across all management levels, some inbred lines such as L6 and L7 combined positive GCA effects for GY and most secondary traits under optimum and managed LN conditions. Similarly, L8 and L9 were identified for good GCA effects for GY under stress conditions. This indicates that Zn donors were adapted to specific environments and thus selection should be based on specific adaptation. Such Zn-enhanced lines could be used as testers or to form synthetics in biofortification programs. However, the locally developed testers were less affected by environmental changes. This shows evidence of the progress made by CIMMYT in breeding for multiple stress tolerance in maize to withstand harsh growing conditions in SSA. However, lines that showed consistency in contributing for low nitrogen and drought tolerance could be further evaluated for nitrogen and water use efficiency. High yielding zinc-enhanced hybrids with specific combinations were identified and could be commercialized in Zimbabwe. Our results demonstrates that some biofortified maize hybrids can be comparable to normal maize in terms of grain yield potential as opposed to previous studies reporting strong dilution effects, grain yield versus nutritional quality.

## Data availability statement

The original contributions presented in the study are included in the article/**Supplementary Material**. Further inquiries can be directed to the corresponding author.

## Author contributions

Conceptualization, NM and ML. Investigation, NM. Resources, TN and ML. Writing–original draft preparation, NM. Writing–review and editing, TN, CK, AB, and ML. Data analysis, CK and NM. Supervision, ML, TN, and AB. Funding acquisition, TN and ML. All authors contributed to the article and approved the submitted version.
