# Supplementary material for: Combining ability and testcross performance of multi-nutrient maize under stress and non-stress environments
Source: Front Plant Sci. 2023 Jan 24;14:1070302. doi: 10.3389/fpls.2023.1070302 (PMC9902879; doi:10.3389/fpls.2023.1070302)
Supplement: Supplementary file 1 [file Table_1.pdf]

**Supplementary Table 1: Ranking of genotypes for grain yield performance (t ha$^{-1}$) under stress and non-stress conditions.**

| Entry (E) | GY OPT | Entry | GY WW | Entry | GY HMDS | Entry | GY LN |
|---|---|---|---|---|---|---|---|
| **84** | 12.35 | **47** | 8.88 | **13** | 4.87 | **28** | 3.31 |
| **5** | 11.34 | **63** | 8.82 | **73** | 4.80 | **35** | 3.20 |
| **82** | 11.24 | **36** | 8.78 | **28** | 4.74 | **11** | 3.20 |
| **83** | 10.65 | **7** | 8.61 | **79** | 4.67 | **21** | 3.15 |
| **35** | 10.14 | **4** | 8.59 | **7** | 4.59 | **78** | 3.14 |
| **62** | 10.04 | **21** | 8.56 | **47** | 4.58 | **17** | 3.09 |
| **38** | 9.84 | **58** | 8.51 | **83** | 4.58 | **18** | 3.08 |
| **80** | 9.79 | **74** | 8.47 | **4** | 4.43 | **10** | 3.07 |
| **6** | 9.65 | **2** | 8.39 | **74** | 4.40 | **83** | 3.03 |
| **10** | 9.63 | **69** | 8.37 | **11** | 4.30 | **5** | 3.03 |
| **4** | 9.49 | **84** | 8.29 | **49** | 4.25 | **26** | 2.97 |
| **33** | 9.43 | **26** | 8.25 | **26** | 4.24 | **54** | 2.94 |
| **1** | 9.38 | **35** | 8.19 | **35** | 4.24 | **7** | 2.91 |
| **69** | 9.36 | **77** | 8.14 | **54** | 4.22 | **3** | 2.85 |
| **39** | 9.34 | **57** | 8.11 | **27** | 4.21 | **47** | 2.76 |
| **12** | 9.29 | **28** | 8.11 | **68** | 4.10 | **75** | 2.73 |
| **23** | 9.15 | **37** | 8.10 | **2** | 4.05 | **82** | 2.69 |
| **13** | 9.11 | **55** | 8.01 | **42** | 4.04 | **19** | 2.66 |
| **9** | 9.09 | **40** | 7.99 | **78** | 4.01 | **68** | 2.51 |
| **28** | 9.08 | **5** | 7.97 | **82** | 4.00 | **84** | 2.50 |
| **68** | 8.97 | **14** | 7.96 | **56** | 3.99 | **61** | 2.38 |
| **79** | 8.80 | **83** | 7.91 | **38** | 3.98 | **69** | 2.36 |
| **40** | 8.78 | **61** | 7.86 | **58** | 3.88 | **33** | 2.26 |
| **42** | 8.77 | **68** | 7.79 | **62** | 3.82 | **57** | 2.25 |
| **36** | 8.76 | **10** | 7.73 | **21** | 3.82 | **13** | 2.23 |
| **21** | 8.72 | **8** | 7.72 | **37** | 3.77 | **41** | 2.22 |
| **58** | 8.68 | **13** | 7.71 | **31** | 3.74 | **49** | 2.20 |
| **18** | 8.62 | **38** | 7.60 | **33** | 3.67 | **74** | 2.13 |
| **41** | 8.61 | **60** | 7.60 | **17** | 3.61 | **8** | 2.07 |
| **48** | 8.56 | **73** | 7.60 | **3** | 3.56 | **79** | 2.06 |
| **74** | 8.46 | **34** | 7.59 | **76** | 3.55 | **6** | 2.04 |
| **27** | 8.34 | **44** | 7.46 | **22** | 3.54 | **55** | 2.03 |
| **7** | 8.31 | **64** | 7.46 | **48** | 3.48 | **67** | 2.00 |
| **75** | 8.30 | **16** | 7.43 | **69** | 3.46 | **48** | 1.97 |
| **61** | 8.25 | **41** | 7.40 | **84** | 3.45 | **39** | 1.95 |
| **71** | 8.19 | **12** | 7.36 | **60** | 3.42 | **2** | 1.95 |
| **77** | 8.17 | **79** | 7.35 | **19** | 3.38 | **81** | 1.94 |
| **20** | 8.09 | **19** | 7.34 | **23** | 3.36 | **1** | 1.94 |
| **24** | 8.08 | **82** | 7.33 | **71** | 3.34 | **58** | 1.92 |
| **55** | 7.98 | **51** | 7.26 | **55** | 3.34 | **38** | 1.86 |
| **37** | 7.97 | **6** | 7.26 | **14** | 3.30 | **16** | 1.86 |
| **67** | 7.94 | **17** | 7.26 | **24** | 3.28 | **59** | 1.84 |
| **34** | 7.93 | **56** | 7.25 | **51** | 3.24 | **73** | 1.81 |
| **31** | 7.93 | **42** | 7.20 | **43** | 3.23 | **4** | 1.80 |
| **19** | 7.91 | **78** | 7.20 | **25** | 3.21 | **12** | 1.80 |
| **76** | 7.74 | **11** | 7.19 | **65** | 3.14 | **76** | 1.75 |
| **47** | 7.59 | **75** | 7.14 | **64** | 3.14 | **22** | 1.73 |
| **51** | 7.59 | **54** | 7.14 | **59** | 3.14 | **37** | 1.69 |

| | | | | | | | |
|---|---|---|---|---|---|---|---|
| **32** | 7.53 | **30** | 7.08 | **57** | 3.13 | **32** | 1.68 |

GY = Grain yield (t $ha^{-1}$), OPT = optimum, WW = well-watered, HMDS = combined heat and drought, LN = managed low nitrogen conditions.
