# Supplementary material for: Combining ability and testcross performance of multi-nutrient maize under stress and non-stress environments
Source: Front Plant Sci. 2023 Jan 24;14:1070302. doi: 10.3389/fpls.2023.1070302 (PMC9902879; doi:10.3389/fpls.2023.1070302)
Supplement: Supplementary file 2 [file Table_2.pdf]

Supplementary Table 2

Environments	Year	Season	Management	Statistics	Traits	
					Zn	Fe
**CIMMYT**	2019	Summer	Optimum	Mean	30.7	29.7
				Range	12.9–46.7	11.3–55.1
				SD	6.4	8.2
**ART Farm**	2019	Summer	Optimum	Mean	29.9	30.1
				Range	16.4–49.2	12.2–54.9
				SD	5.8	7.91
**DR&SS**	2019	Summer	Managed Low N	Mean	24.8	20.4
				Range	13.9–39.5	7.81–34.9
				SD	5.2	6.1
**CHISUMBANJE**	2020	Summer	Heat and Drought	Mean	26.2	23.0
				Range	10.7–39.4	7.1–37.6
				SD	5.1	4.9
**RARS**	2019	Summer	Optimum	Mean	30.5	29.9
				Range	17.3–45.3	12.6–58.4
				SD	5.9	8.1
**GWEBI**	2019	Summer	Optimum	Mean	30.4	30.8
				Range	15.6–57.8	12.5–54.4
				SD	6.3	7.7
**CHISUMBANJE**	2020	Winter	Well-Watered	Mean	25.2	23.1
				Range	12.4–36.6	9.2–37.1
				SD	4.9	6.1
**CHIREDZI**	2020	Winter	Heat and Drought	Mean	25.1	21.1
				Range	12.1–36.8	7.9–42.8
				SD	5.2	6.1
**CHIREDZI**	2020	Winter	Well-Watered	Mean	28.3	31.5
				Range	13.3–38.5	10.5–34.9
				SD	4.7	6.5
**CIMMYT**	2019	Summer	Managed Low N	Mean	23.5	22.6
				Range	16.4–34.8	9.7–33.8
				SD	3.5	4.4

Zn = grain zinc concentration (mg kg^{-1}); Fe = Grain iron concentrtion (mg kg^{-1}), SD = Standard deviation.
